# A remarkable new genus of leafhoppers (Hemiptera, Cicadellidae, Iassinae) from Southeast Asia

**DOI:** 10.3897/zookeys.239.3960

**Published:** 2012-11-08

**Authors:** Wu Dai, Chris H. Dietrich

**Affiliations:** 1 Key Laboratory of Plant Protection Resources and Pest Management, Ministry of Education, Entomological Museum, Northwest A&F University, Yangling, Shaanxi 712100, China; 2 Illinois Natural History Survey, Prairie Research Institute, University of Illinois at Urbana-Champaign, 1816 S. Oak St., Champaign, IL 61820

**Keywords:** Homoptera, Auchenorrhyncha, morphology, distribution, taxonomy

## Abstract

*Tardrabassus pakneunensis*, **n. gen. & sp.** is described and illustrated. The new genus shows morphological affinities to three leafhopper subfamilies, Tartessinae, Deltocephalinae, and Iassinae, but is tentatively placed in Iassinae based on the position of the ocelli, the reduced lateral frontal sutures, the leg chaetotaxy, and the structure of the male genitalia.

## Introduction

The higher classification of leafhoppers, family Cicadellidae, has long been controversial, partly due to the tremendous diversity of the family (>20,000 described species in >2,600 genera) and the fact that there are numerous genera that have combinations of the morphological features normally diagnostic for more than one subfamily or tribe (e.g., Wei et al. 2007, 2010, Dietrich 2011a,b; Viraktamath and Dietrich 2011). Such taxa are important because they may bridge morphological gaps between major leafhopper lineages and shed light on phylogenetic relationships. The new leafhopper species described herein falls into this category. Here we describe and illustrate the new species, place it in a new genus and include it tentatively in the cicadellid subfamily Iassinae.

## Materials and methods

Specimens examined are deposited in The Natural History Museum, London (BMNH). Morphological terminology follows Dietrich (2005).

## Taxonomy

### Subfamily Iassinae

#### Tardrabassus gen. n.

urn:lsid:zoobank.org:act:EDAD4D82-8911-4713-96BF-AA3F624D6CE0

http://species-id.net/wiki/Tardrabassus

##### Type species:

*Tardrabassus pakneunensis* sp. n.

##### Description.

Robust, depressed leafhoppers. Color mostly dark brown to black with few symmetrical yellow markings.

**Figure 1.**
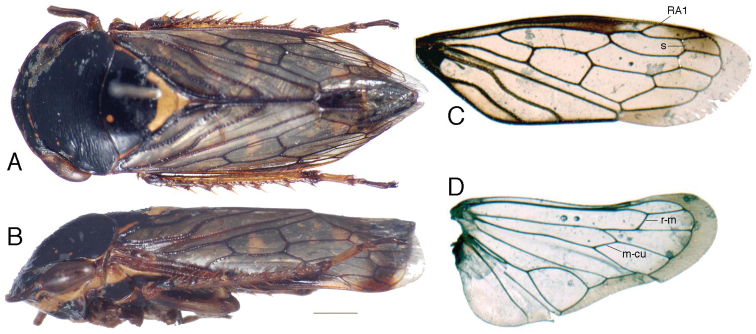
*Tardrabassus pakneunensis* sp. n. **A** habitus, dorsal view **B** same, lateral view **C** forewing **D** hind wing. Scale bar = 1 mm.

**Figure 2.**
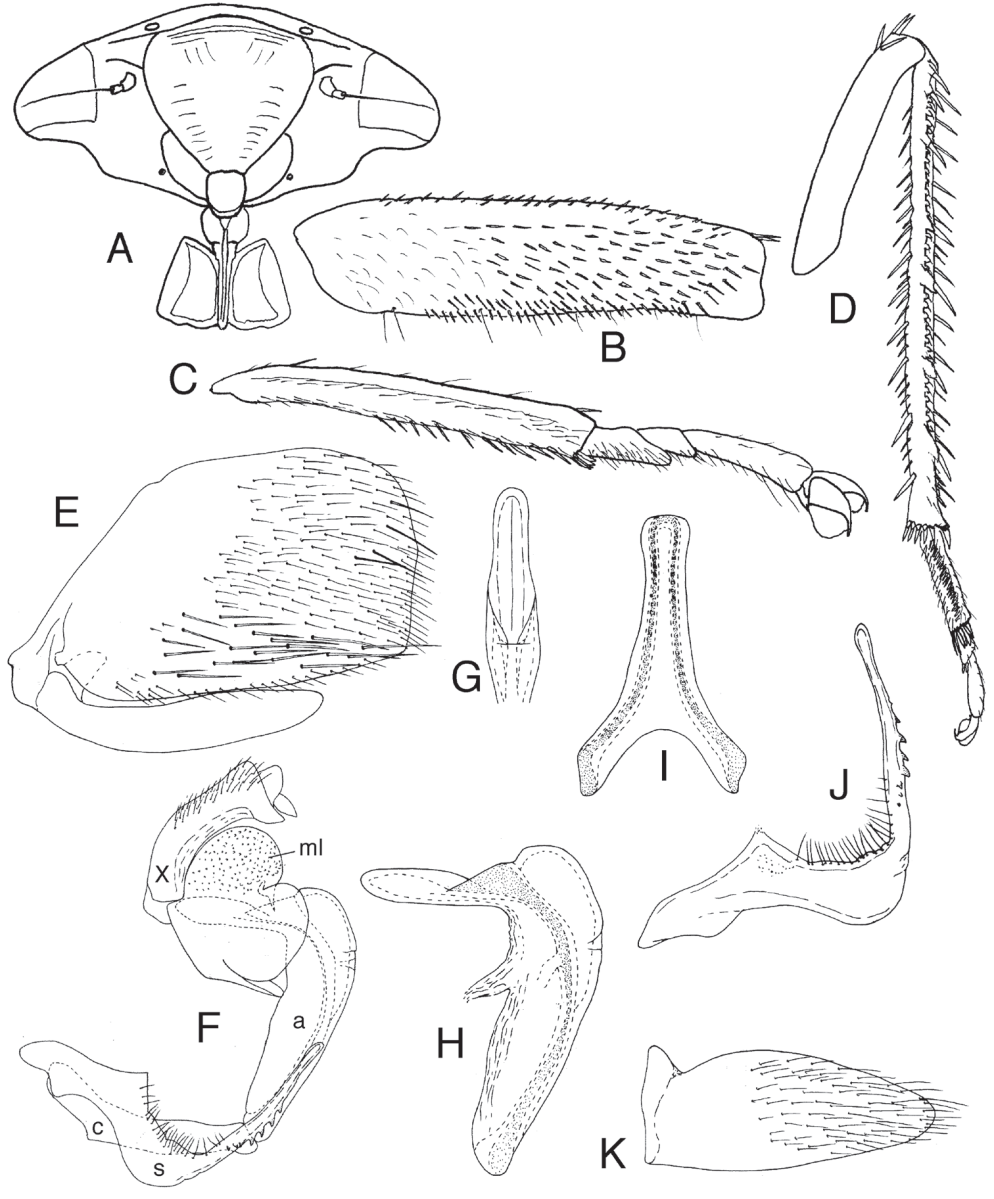
*Tardrabassus pakneunensis* sp. n. **A** head, anteroventral view **B** front femur, anterior view **C** front tibia and tarsus, anterior view **D** hind leg (except coxa and trochanter), anterior view **E** male pygofer and subgenital plate, lateral view (chaetotaxy of plate not shown) **F** male genitalia and anal tube, lateral view: anal tube (**X**), membranous internal lobes of genital capsule (**ml**), aedeagus (**a**), connective (**c**) and style (**s**) **G** apex of aedeagus, caudal view **H** aedeagus, lateral view **I** connective, dorsal view **J** style, lateral view **K** left subgenital plate, ventral view.

Head in dorsal view (Fig. 1A) wider than pronotum; crown short, more than four times wider than long, anterior and posterior margins parallel; texture longitudinally rugulose and with numerous minute pits; with indistinct transverse depression preapically; ocelli small, on crown just posterad of anterior margin, mesad of antennal pits and well separated from eyes; anterior margin of head depressed, forming distinct shelf in lateral view (Fig. 1B), transition from crown to face narrow but smooth, without transverse carina; frontoclypeus in anterior view (Fig. 2A) evenly broadened from anteclypeus to dorsal margin, convex and rugulose ventrally, concave and transversely striate dorsally; antennal ledge closer to anterodorsal than to anteroventral corner of eye, represented by prominent carina, slightly oblique, nearly horizontal, not concealing antennal base; antenna length one third width of head; lateral frontal sutures extended dorsomesad from antennal pits to margin of crown, not reaching ocelli; gena broad, obtusely incised ventrad of eye, with obtuse ventrolateral projection almost completely concealing proepisternum; lorum large, flat, dorsal 2/3 bordering frontoclypeus, ventral 1/3 bordering anteclypeus, extended nearly to ventral margin of gena; maxillary sensillum near mid-height of lorum, closer to lorum than to lateral margin of gena; anteclypeus small slightly convex, ovoid, with transverse preapical concavity, apical margin carinate, rounded and slightly upturned, extended slightly beyond ventral margin of gena; rostrum with distal segment greatly expanded and depressed, lamelliform.

Pronotum depressed, anterior margin produced but not extended anterad of eyes medially (Fig. 1A), posterior margin weakly concave, transverse rugae well developed, lateral margin shorter than eye, carinate. Exposed part of mesonotum and scutellum as long as pronotum; mesonotum rugulose; scutellum transversely striate, apex acuminate. Forewing (Fig. 1C) smoky hyaline throughout length except small opaquely sclerotized area along costal margin, with minute setae and pits present on claval veins and on veins in basal third of corium; appendix very broad, crenulate, extended around wing apex; vein RA1 arising distad of RA-RP fork; crossvein s present; crossvein r-m1 connected to RP; three m-cu crossveins present; brachial cell narrow, parallel sided; CuA connected to submarginal vein slightly distad of clavus apex; inner apical cell long, maximum width subequal to that of apical cell 2; Pcu and A1 sinuate. Hind wing venation complete (Fig. 1D); RP and MA free, connected by crossvein; m-cu long and oblique; costal margin not humped near base; wing margin beyond submarginal vein wide; submarginal vein not extended onto jugum. Front femur (Fig. 2B) anterior surface with numerous scattered, poorly differentiated setae, AV with few long, fine setae basally; tibia (Fig. 2C) with dorsal surface flattened but not expanded, dorsal rows undifferentiated, ventral rows with few stout preapical macrosetae. Hind femur (Fig. 2D) with setal formula 2+2 with penultimate pair well separated; tibial row PD with alternating short and long macrosetae, AD with macrosetal bases spinelike, with 4-8 cucullate intercalary setae between successive macrosetae, AV with ~7 macrosetae and 2-5 cucullate intercalary setae between successive macrosetae, PV with numerous long tapered setae, pecten with 2 transverse rows of spines, distal row with macrosetae alternating short-long; tarsomere I long, without dorsoapical macrosetae or ventral heel, rows AV and PV differentiated but irregular, pecten with 2-3 platellae laterally and 3-4 tapered pale setae medially; tarsomere II less than 1/3 length of tarsomere I.

Male abdomen with tergite I transverse, acrotergite small, elliptical; ventral apodemes absent. Sternite VIII longer than sternite VII, posterior margin roundly produced, concealing ~2/3 of subgenital plate in repose. Valve very short, straplike, narrowly fused to pygofer (Fig. 2E). Subgenital plates (Figs 2E, K) ligulate, strongly depressed, medial margin straight; lateral margin rounded, widened to 1/3 length, thence narrowed to bluntly rounded apex; with numerous scattered setae of various sizes ventrally, more densely distributed toward lateral margin. Pygofer (Fig. 2E) with tergite short, bandlike, with short dorsal and ventral clefts at base of lateral lobes; lobes large, quadrate, extended well beyond subgenital plate apex, without processes, with numerous scattered setae of various sizes distributed in distal ¾. Anal tube (Fig. 2F) moderately long, well sclerotized dorsally, without processes. Style (Fig. 2J) apodeme massive, expanded apically, extended dorsad at right angle to apophysis; basolateral lobe large, rounded; apophysis elongate, slender, with several prominent ventral preapical teeth, apex slightly expanded and blunt. Connective (Fig. 2I) large, well sclerotized, Y-shaped, stem longer than arms. Aedeagus (Figs 2G–H) with atrium large, with median longitudinal ventral carina; shaft L-shaped, tubular, tapered distally, gonopore apical on anterior surface. Interior membrane of genital capsule (Fig. 2F) with two pairs of large, partially sclerotized lateral lobes enclosing aedeagal shaft, dorsal pair with numerous conspicuous microtrichia; pair of sclerotized vertical flaps articulated basad of lobes extended between aedeagus and base of anal tube.

##### Distribution.

Southeast Asia (Laos).

##### Etymology.

The genus name, a masculine noun, was formed by combining the names of three genera that resemble the new genus in certain respects: *Tartessus*, *Drabescus*, and *Iassus*, reflecting the apparently mixed morphological affinities of the genus.

##### Notes.

*Tardrabassus* is difficult to place in the present subfamily classification of leafhoppers. The structure of the head somewhat resembles that of *Drabescus* (Deltocephalinae: Paraboloponini) in coloration and in the form of the antennal ledges and shape of the facial sclerites. It also resembles *Tartessus* (Tartessinae) in having cucullate intercalary setae on hind tibial row AD. It resembles both of these genera in the form of the pronotum (produced medially with prominent transverse rugae) and forewing (broad appendix extended around wing apex, venation complete and well delimited). The placement of the ocelli on the crown, the dorsally obsolete lateral frontal sutures, and the poorly differentiated chaetotaxy of the front femur distinguish the new genus from *Drabescus* and most Tartessinae (except some Thymbrini--recently transferred from Ledrinae by Jones and Deitz (2009)), but are consistent with both Iassinae and Ledrinae. Ledrinae have the ocelli on the crown, but distant from the anterior margin and have the ocellocular area of the face much wider. In the structure of the male genitalia, however, *Tardrabassus* is most similar to Iassinae. The pregenital sternite is enlarged with the posterior margin produced, concealing the basal half of the subgenital plates when the genital capsule is retracted, a synapomorphy of Iassinae. Other aspects of the male genitalia, including the ligulate subgenital plates, the sigmoid style, and the simple aedeagus are all plesiomorphic features shared with various other leafhopper groups. The unusual combination of plesiomorphic (e.g., poorly differentiated front femoral chaetotaxy, presence of an r-m crossvein in the hind wing) and apomorphic features (e.g., retracted male genital capsule, broad gena) suggests that *Tardrabassus* represents either an early diverging lineage of Iassinae or a lineage distinct from previously recognized cicadellid subfamilies. Among previously described genera of Iassinae it is perhaps most similar to the Neotropical genus *Bythonia* (Bythoniini), but differs in having the ocelli on the crown, the antennal ledges and lateral frontal sutures reduced, and the dorsum without conspicuous setae.

The distal segment of the rostrum of *Tardrabassus* is more strongly expanded than in any other known leafhopper. Similar, although less extreme, conditions occur in *Coriojassus* Evans (Iassinae) and in various Idiocerini. The shape of the distal segment varies among the three pinned males available for study, possibly because the expanded part is partially membranous and became distorted as a result of drying. In other leafhoppers that exhibit a similar condition, it occurs only in males. We cannot confirm that the condition is sexually dimorphic in *Tardrabassus* because females remain unknown. The function of this modification is also unknown.

#### Tardrabassus pakneunensis sp. n.

urn:lsid:zoobank.org:act:9F23AB27-E2CD-4D39-B5AF-B51A5A5D6182

http://species-id.net/wiki/Tardrabassus_pakneunensis

Figs 1 2

##### Type locality:

Pak Neun, Luang Prabang, Laos [BMNH].

##### Description.

Length of male 10.0–10.8 mm. Head with anterior margin narrowly orange-brown, crown with few small symmetrical orange spots, face with broad transverse yellow band extended across middle of frontoclypeus, continuing onto thoracic pleuron and extended to mesepimeron; anteclypeus yellow with brown medial spot; scutellum with posterolateral margins yellow; forewing hyaline, veins brown. Aedeagus without processes, with posterior convexity near base of shaft in lateral view.

##### Material examined.

Holotype male labeled “Luang Prabang/ Pak Neun./ 28.IX.1918/ R.V. de Salvaza.; Indo China./ R. V. de Salvaza./ 1918-1”; one male paratype, same data; one male paratype, [LAOS?]: Huat Mekong, Pang Ngeon, 15 V 1918, R. V. de Salvaza [BMNH].

##### Notes.

The species name is based on that of the type locality, a village on the Mekong River in northern Laos. “Pang Ngeon” could not be located in available gazetteers and databases but may represent an alternative spelling of the type locality. Additional specimens have not been found, despite extensive recent field work in Thailand and Vietnam.

## Supplementary Material

XML Treatment for Tardrabassus

XML Treatment for Tardrabassus pakneunensis
